# Improving welfare assessment in aquaculture

**DOI:** 10.3389/fvets.2023.1060720

**Published:** 2023-02-28

**Authors:** Heather Browning

**Affiliations:** ^1^Center for Philosophy of Natural and Social Science, London School of Economics and Political Science, London, United Kingdom; ^2^Department of Philosophy, University of Southampton, Southampton, United Kingdom

**Keywords:** aquaculture, fish, welfare, assessment, certification

## Abstract

While global aquaculture is rapidly expanding, there remains little attention given to the assessment of animal welfare within aquacultural systems. It is crucial that animal welfare concerns are central in the development and implementation of aquaculture as if they are not prioritized early on, it becomes much more difficult to adapt in future. To this end, it is important to ensure the availability of high-quality welfare assessment schemes to evaluate the welfare of animals in aquaculture and promote and maintain high welfare standards. This paper will first discuss some of the current certification and assessment frameworks, highlighting the primary limitations that need to be addressed, before going on to describe the recommendations for a best-practice welfare assessment process for aquaculture; with the hope that these considerations can be taken on board and used to help improve welfare assessment for aquaculture and, ultimately, to ensure animals used in aquaculture have a higher level of welfare. Any aquacultural system should be assessed according to a suitable framework in order to be considered adequate for the welfare of the animals it contains, and thus to maintain social license to operate.

## 1. Introduction

Aquaculture is a rapidly expanding industry, with current estimates suggesting there are over 100 billion fish produced annually.^1^ This expansion is set to continue, with the recent Blue Foods movement^2^ pushing for a global expansion of aquaculture. While there are many potential issues with this movement, such as human health and environmental sustainability, one of the biggest problems it raises is of the welfare of the animals used in aquaculture. Importantly, the issue of welfare is currently given very little attention in the literature on Blue Foods production [e.g., (1)], but should play a much more central role in assessing the ethics and sustainability of aquacultural systems. Good welfare should be one of the core goals of the Blue Foods movement and of aquaculture more generally. The primary goal of this paper is to emphasize the importance of welfare considerations in the development and implementation of aquaculture, while demonstrating some of the primary shortcomings that need to be addressed for better welfare assessment moving forward. While the principles and recommendations described are not specific to aquaculture, I take them to be particularly relevant in this context due to the lacks in the current methods used.

Most animals used in aquaculture are finfish (e.g., salmon, trout).^3^ There has been much debate regarding whether fish are sentient—i.e., capable of experiencing feelings of pleasure or suffering—primarily due to differences in neural anatomy that may or may not be sufficient for conscious experience (3–9). However, evidence of their complex perception, cognition, learning, and behavioral responses, has led many to conclude they are sentient (10–16) and thus have a welfare that can be harmed by poor housing or husbandry practices. Precautionary reasoning suggests that this evidence should still be taken as sufficient for treating animals as sentient, to avoid causing inadvertent harm, even if certainty is lacking (17). With the large number of animals currently produced through aquaculture, and the even higher numbers predicted for the future, this is clearly a consequential issue. There are many aspects of commercial aquaculture that are likely to lead to poor welfare, such as poor water quality, overcrowding, starvation, disease, predation, exposure to environmental stressors, restricted behavior, and improper handling, transport, and killing (18, 19).

Part of considering animal welfare means being clear about what is meant by welfare. There are several commonly-used definitions of animal welfare—biological functioning (physical health and fitness), natural living (performance of natural behaviors), preferences (having what the animal wants), and affective state (the animal's feelings, or subjective experience). Some frameworks will use multiple components, such as the “tripartite” concept of feeling good, functioning well, and living naturally (20, 21), or Dawkins' “two questions” approach that looks at whether an animal is healthy and has what it wants (22, 23). There are also strong links between many of these aspects—for instance the ways feelings may guide animal preferences and behavioral motivation (24) or that evolved natural behaviors will often feel good and be strongly motivated (25)—and this can lead to disagreements about which component is primary. However, while there is still no widespread agreement about which of these, or which grouping of them, is best to use, it is becoming increasingly common for definitions of welfare to include affective state as at least part of what counts in defining and assessing welfare [for further discussion and defense of this view, see (26, 27)]. This is linked to the widespread idea that it is the capacity for suffering (and pleasure) that matters morally and grounds our ethical concern for animals in the first place. Accounts that lack this component risk disengaging themselves from the ethical relevance of welfare (28). Whether one takes the feelings-based account, or the more comprehensive multi-component account, it is clear that it is important to ensure that a welfare assessment takes into account the psychological wellbeing of the animals, not just their physical health.

As part of this, it is important also to consider not only potential negative feelings, but also positive, as welfare should be considered as a spectrum from good to poor. While traditional animal welfare science has focused on measurement and prevention of suffering, there is increasing recognition that the positive states of welfare are also important (29, 30). Positive feelings relevant to welfare could include comfort, satiation, sensory pleasures, social bonds, or the pleasures accompanying behaviors such as exploration or play. While different animals are likely to differ in exactly which of these feelings they can or do experience, they should not be overlooked when thinking about subjectively experienced welfare.

It is important that aquacultural systems ensure good welfare of the animals in their care. However, this can only be done through performing regular welfare assessments using valid indicators of fish welfare, to ensure the conditions of care are suitable, and the animals are doing well. Importantly, we can't ensure that animals have good welfare without having any means to monitor or assess it. Unfortunately, there are no current ongoing reliable methods of welfare monitoring in fish aquaculture (18). While there are currently several certification systems in use for aquaculture, welfare plays a relatively small part in almost all of these. What is needed is a more comprehensive welfare-focused assessment system that can be applied uniformly, to regulate animal welfare in aquaculture, and help inform consumer decisions. This paper aims to help provide some of the guiding principles that should underlie development of such a system. While these are applicable to all types of animal management systems, not just aquaculture, they are focused on where aquaculture in particular is currently lacking.

This paper will first discuss some of the current certification and assessment frameworks, highlighting their limitations, before going on to describe some recommendations for a best-practice welfare assessment process for aquaculture, such as providing a complete assessment of welfare (including valid measures of affective states), feasible for use within an aquaculture setting, and setting appropriate threshold levels for what counts as acceptable welfare. It is not my intention here to create or provide the details of such a framework, but rather to provide recommendations from which one can be created, or by which existing or future welfare assessment tools can be evaluated. The hope is that these considerations can be taken on board and used to help improve welfare assessment for aquaculture and, ultimately, to ensure animals used in aquaculture have good welfare. As is best practice for all systems of animal use, any aquacultural system should be assessed according to a suitable framework in order to be considered adequate for the welfare of the animals it contains, and thus to maintain social license to operate.

## 2. Current practice

### 2.1. Certification schemes

There are a variety of different certification programs used for aquaculture. Some of the more prominent of these include the Global Seafoods Alliance Best Aquaculture Practices (BAP),^4^ the Global Agricultural Practices Aquaculture certification,^5^ the Aquaculture Stewardship Program,^6^ and the UK's RSPCA Assured program.^7^

One of the first problems is that almost all of these programs have welfare as only one small part, alongside other considerations such as environmental impact, social impact, and food safety. Only the RSPCA Assured program focuses on welfare. The RSPCA standards are guidelines for the housing and husbandry of salmon (31) and rainbow trout (32) which describe the requirements for a range of management and environmental conditions for the animals, including the on-farm procedures, handling, transport, health management, water quality, feeding, and slaughter. They also list some “welfare assessment outcomes” (such as skin, deformities, eyes, abnormal behavior, and mortality rates) but say these are currently not formalized.

Secondly, it is difficult to find the details on most of these standards. Even where the specific certification requirements are provided, they often refer to “appropriate” or “adequate” conditions, or “minimal” or “unnecessary” stress and/or suffering, without specifying exactly what these are. There is thus a concern that this will mean different things in different contexts, lacking regularity across the industry, or that they can be set arbitrarily according to the interests of producers.

Finally, the biggest limitation of these programs is that they are formed almost entirely of input measures.^8^ It is common to divide measures of animal welfare into two categories—often called “input” and “outcome” measures, “environment” and “animal”, or “causal” and “effect” (33). Input measures are welfare indicators that measure some conditions within the environment that will have an impact on the physical or psychological state of the animal—examples include water quality, nutrition, and stocking density. These are then causal inputs to the state of welfare in the animals. Outcome measures are welfare indicators that track the changes in physiology and behavior of the animal in relation to its welfare state, such as poor health, or aggression. These are thus the effects of inadequate living conditions, or poor welfare.

Input measures are often used as they can be easier to collect—simply requiring measurement of pen sizes, temperature, or water sampling. However, they are limited in that they will only work well when a full set is present. For example, measuring only water quality will not tell us much about fish welfare if we don't know anything about the quality of their nutrition. These measures are often taken to assess potential risks to welfare, rather than as measures of welfare itself (34).

By contrast, outcome measures provide a more direct picture of the welfare of the animal, telling us how it is responding to the conditions in its environment. These measures can be more difficult or costly to collect, however. Where these measures are included within the certification schemes, they are only health-based measures, without consideration of the importance of mental states for welfare, as will be discussed further in the next section.

### 2.2. Assessment frameworks

There are a number of welfare assessment frameworks for aquaculture. The primary of these is the SWIM (salmon welfare index) model (35, 36), which has been built for salmon, but also adapted for sea bass (37) and lumpfish (38). There are also the FISHWELL models, for salmon and trout (39, 40), and the MyFishCheck system (41) which is intended as a more general model, with an app for use on farms. These systems have the benefit of being entirely welfare-focused, using a range of indicators for different aspects of welfare to output an overall welfare score. Importantly, they bring together both input and outcome measures, grouped around different welfare “needs”. These needs included physical needs such as respiration, osmotic balance, nutrition, and health, and behavioral needs such as feeding, social contact, and exploration, and were measured by a wide range of indicators. For example, the SWIM model uses environmental indicators such as water temp, salinity, density, and animal-based indicators including: mortality, appetite, sea lice, body condition, and fin/skin damage. The FISHWELL system focuses more strongly on animal indicators including emaciation, skin damage, scale loss, eye problems, gill problems, deformities, sea lice, fin damage. They also provide a set of context-specific environmental indicators for different management systems. MyFishCheck is divided up into modules covering farm management (e.g., personnel training, cleanliness, procedures, record keeping, feeding, lighting, stocking density), water quality (temp, O2, ammonia, etc.), fish group behavior (aggression, ventilation rate, isolation, fleeing, feeding), fish external appearance (deformations, injuries, body condition), and fish internal appearance (organs, physiology, pathogens).

These frameworks have the advantage of being entirely welfare focused. However, there are also several limitations with these systems. The first is the question of how scores are aggregated into a final grade, particularly how different components are weighted. This is a well-known problem within multi-criteria welfare frameworks such as these (42). Weightings can be highly subjective, and the procedures often opaque. The SWIM model acknowledges this and makes an attempt to be objective about weightings through a thorough literature review that allows a “somewhat subjective, but systematic, scoring based on an assessment of the intensity, duration and incidence of the welfare impact as implied by each scientific statement that has been linked to the [welfare indicator]” [(36), p. 35]. While this is the best currently available method, as the authors acknowledge, there is currently insufficient information available to reliably set weights.

The second problem is that they provide an incomplete picture. That is, while they do well at measuring physical health, they have little consideration of psychological wellbeing. While there are some mentions in the SWIM model of welfare needs such as social contact, and exploration, these are taken to be covered by environmental indicators, without direct validation that these indicators really do track fulfillment of these needs (the issue of validation will be discussed further in the next section). Fishwell describes behavioral measures, but its scoring scheme leaves them out. MyFishCheck includes a few behavioral measures intended to indicate poor psychological welfare (e.g., “apathy”, “fleeing”) but these are not well-defined and form only a small portion of the total assessment. More generally, this issue of selection of appropriate welfare indicators to provide a complete picture of total welfare experience relates back to the choice of definition of welfare. I have already discussed the different concepts of welfare and the importance of considering animal feelings alongside health or physical functioning. While good health is certainly necessary for good welfare, it is not sufficient on its own, without some consideration of the psychological needs of the animals, whether considered as behavioral needs, feelings, or what the animal wants. While many welfare assessment frameworks used for land-based agriculture—such as the Welfare Quality assessments commonly used for several species within the EU (43)—now explicitly include measures aimed at assessing feelings, or psychological wellbeing, its absence in aquaculture is notable and suggests that practitioners within the aquacultural context are focusing only on the physical or health-based aspects of welfare. Where the models do nominally describe behavioral needs, these are then operationalized only in terms of broad environmental provisions, rather than assessing the effects from the point of view of the fish.

Finally, there is the issue of setting appropriate thresholds. This applies to both the certification schemes and these welfare assessment frameworks. All of these have to set the levels at which a measure (or the set of measures) indicate that the animal has a poor, acceptable, or good level of welfare. These can be set for individual indicator scores (e.g., number of skin lesions) or for housing standards (e.g., stocking density). For a certification scheme, a certain minimum standard must be met in order to pass the checks and receive certification. For the assessment frameworks, it is not essential to set such levels, as a score can be assigned regardless of threshold, but it is common to do so—for instance, the SWIM model assigns threshold levels of welfare impact for each indicator (36) and in some uses takes the highest overall score to represent “good” welfare (37). However, the setting of thresholds for acceptable welfare is not an empirical process. It is instead a value-based decision, regarding what we think of as acceptable levels of welfare, and thus can be highly subjective.

It is thus important that there is input from multiple stakeholders in determining what should count as an acceptable welfare threshold. This could be accomplished through something like a Delphi process, in which a group of experts can discuss and refine their decisions to reach agreement (44–47). These should definitely not be set by industry alone, relative only to the conditions regularly seen on-farm—in this case, it could end up that if all farms are not good, then even the highest welfare scores would not actually represent good welfare. Take, for example, one of the on-farm tests of the SWIM model (48). Here, the scores are scaled such that 0 represents the lowest, and 1 the highest score. However, in their 19 assessments (10 farms, all but one visited twice), all received scores above 0.5, and several above 0.8. Given what we know about the range of potential welfare issues in salmon farming, it is highly unlikely that a substantial portion of farms provide salmon with welfare so exceptional it is at 80% of their maximum possible welfare experience. It is far more likely that this is set relative to industry standards, such that these farms do well for a farm but not necessarily for the fish.

Once these thresholds are set, there is then an additional project in determining, for each fish species of interest, what the appropriate housing and husbandry conditions are for meeting this threshold. This project would involve ongoing testing of fish under different conditions, using a range of indicators, such as measures of health, and of the preferences of the animals for different environments or experiences.

Also relevant here is appropriate consideration of positive welfare. As discussed, good welfare should not simply be considered as the absence of suffering or negative affect, but also the presence of positive experiences. This means that when setting thresholds for acceptable levels of welfare, this should not be based only on a “neutral” level of welfare, where all suffering is presumed to be removed, but also with some positive experiences promoted. While the provision of appropriate housing and husbandry to ensure positive experiences for fish is beyond the scope of this paper on welfare measurement,^9^ I do want to also emphasize that this can't be achieved without methods for measuring or identifying positive welfare states. Any welfare assessment should also have room to identify the presence of positive welfare states, and these should be incorporated into minimum welfare standards.

While the existence of any attempts to measure and certify welfare standards should be applauded, as shown here, there are some strong limitations on the current methods of welfare assessment in aquaculture. The next section will look at how these might be overcome, and what a more ideal welfare assessment framework might look like.

## 3. Recommendations

Based on the considerations discussed above, we are now in a position to consider some recommendations for best practice welfare assessment in aquaculture. The principles I will discuss are not specific to aquaculture—they are relevant to best practice welfare assessment for any animal species. However, those I have chosen here are those that are particularly relevant within the context of aquaculture, as they are those that have been overlooked within the design of the current welfare assessment frameworks and certification programs. A good welfare assessment tool should consider completeness, validity, feasibility, and setting of reasonable thresholds for acceptable welfare.

The first consideration is completeness. As has been discussed, this is one of the primary drawbacks to the existing methods. While we currently lack methods for determining whether any set of indicators is entirely complete [though see suggestion in (42)], they can be assessed for any obvious gaps or failure to cover some aspect of welfare or important condition for welfare. This should involve including both input and outcome measures, to identify the state of the housing and husbandry of the animal (allowing identification of potential improvements), and to determine the actual impact of these conditions on the welfare of the animals. It should also include measures of both physical health and affective state, so that the psychological wellbeing of the animal is part of the picture, accounting for the positive and negative feelings that contribute to welfare. This could also potentially include some measures of opportunities for natural behavior, since, as discussed earlier, this is another commonly used component of welfare, and—importantly for certification programs—one that many consumers seem to endorse (51, 52). Finally, it should include measures representing positive welfare, to allow for and encourage welfare experiences above the neutral baseline. Some possible behavioral measures of positive welfare in fish include feed anticipation and opportunities for exploratory behavior (53). Preference and motivation testing could additionally be used to identify the conditions and experiences the fish want and are likely to experience positive affects with—for example studies showing that cichlid fish value presence of tank substrate, but less than food and access to social companions (54, 55).

When thinking about completeness, it is useful to draw a distinction between partial and whole-animal measures of welfare (42). Partial indicators are those indicators that assess some aspect of or contributor to welfare, and are therefore incomplete on their own. All input measures, such as tank size or oxygen levels, are partial measures; as are many output measures, such as physiological indicators of stress, growth rate, and behavioral changes. I will not here discuss any specific welfare indicators in detail, but there is a growing literature to draw on regarding different indicators that might be appropriate for fish welfare [see, e.g., reviews in (18, 19, 39, 40, 53, 56, 57)].

Some examples of partial indicators include physiological indicators of stress (e.g., gill or scale cortisol, blood glucose), immune system functioning, heart rate, gill ventilation rate, size or growth rate, and condition of skin mucosa. These seem to primarily serve as indicators of fish health and body condition, as well as overall levels of stress. This is fine if they are explicitly used to measure only these contributors to welfare, alongside other measures aimed at the affective states of the fish. While measures of stress have some ability to track psychological experience alongside physiological stress, they cannot differentiate between positive and negative states of arousal (22) and thus must be used alongside other measures that can. There is therefore a key knowledge gap in developing and validating measures that track fish affective states as well as physical health. Behavioral and cognitive partial measures include aggression, feeding behavior, swimming behavior, feeding behavior, exploratory behavior, and stereotypic behaviors. Some of these behaviors can indicate how well a fish is meeting its needs—for example, feeding behavior relates to nutritional needs, and is likely to relate to the experiences of hunger and satiation. Others, such as aggression or stereotypic behavior can indicate past or present welfare problems, such as feelings of frustration or unmet behavioral needs (58).

Partial measures can be used together to form a more complete picture of overall welfare, such as is common in many welfare assessment frameworks used for agricultural animals, including Welfare Quality (43), Five Domains (59), and welfare Decision Support Systems [e.g., SOWEL models; (60, 61)]. However, when this set is inaccurate (contains measures unrelated to welfare) or incomplete (fails to include measures of important aspects of welfare), there will be a loss of information about welfare, and the risk of drawing inaccurate conclusions. Completeness of a set of indicators can be judged by expert panels to pick up on any obvious mistakes or exclusions, such as in the building of the welfare Decision Support Systems (60, 61), or validated against whole-animal measures of welfare (42).

In contrast with partial measures, whole-animal measures use a single measure to represent the entire state of welfare for the animal. This has the obvious benefit of being a complete welfare measure, inclusive of all the external and internal states that are impacting an animal's welfare. Examples of whole-animal indicators of welfare that may work for fish include Qualitative Behavioral Analysis (QBA), cognitive bias, laterality, and skin mucosa. QBA uses an observer assessment of the overall behavioral profile of the animal to form a judgment about its welfare (62, 63), and has shown both good inter-observer reliability and validity when tested against other welfare indicators (64). The method has been successfully used in a variety of mammals and some birds (65–70) and some recent work suggests it will also work in salmon (71). If this method can be further explored and validated for fish, it could form a powerful on-farm method of assessing fish welfare.

Cognitive bias testing looks to measure the overall affective state of an animal through its effects on different cognitive processes, such as attention and memory. The most well-developed is cognitive judgment bias, a method that measures an animal's affective state through the degree to which it demonstrates optimism or pessimism in interpreting ambiguous stimuli (72, 73). This method can be used to assess the overall welfare state of an animal as represented by its “mood”, though it is not yet entirely certain how this relates to other aspects, such as interaction with animal preferences, or acute vs. ongoing welfare experience (74). It has been used across a range of species and welfare conditions, such as differences in environment, feeding, and social housing (72) and has been successfully performed with fish to test for the effects of pair bonding on affective state in a cichlid (75). Its primary drawback is that it requires prior training of the animal in order to administer and thus is less useful for an on-farm assessment. It could perhaps be used instead for a more general test of the welfare of a fish housed in similar conditions within a testing situation, with an inference to the probable welfare of fish on farm. Other types of cognitive bias testing, such as attention bias, require less preparation for testing and may thus be of more use within an assessment scheme (76), but more work is needed to develop these methods for use with any animals, with no current work using it as an indicator for fish.

Measures of laterality rely on the fact that positive and negative emotions are processed in different hemispheres of the brain, and measures that determine the dominant side may thus indicate the current welfare status of the animal (77). A recent review has proposed laterality as a useful welfare indicator for fish (78). However, while there is some evidence laterality will alter with stress and changes in emotional state, these measures require further validation before use—it is currently uncertain whether changes in laterality would track changes in welfare state for fish and in particular whether this relates to affective experience or just stress more generally.

One final potential measure of fish welfare is quality of skin mucosa. Fish skin mucosa is sensitive and will worsen with poor health, poor diet, and stress (79), so could potentially be a good whole-animal indicator; however it currently seems primarily to track changes in health and physiological stress and while these may be related to how the animal feels, more work is needed to validate it as a good measure of affective states.

There are a range of potential whole-animal measures of welfare, although these are all relatively new and require more work to be more confident about their use for fish. Once developed, such measures will be powerful tools as they would allow us to take a single measurement to represent the entire welfare experience of the animal. In the meantime, our next-best option is to use what we have, supported by our best attempts at complete sets of partial measures. As whole-animal measures don't tell us anything about the specific contributors to good or poor welfare, partial indicators can help with making specific recommendations as they can each tell us something about which environmental conditions are influencing welfare, or which specific positive or negative affects the animal is experiencing. These then allow for targeted interventions where they are needed to improve welfare. I have argued elsewhere that for this reason it is best to use whole-animal measures alongside well-constructed sets of partial indicators, to gain the benefits and offset the drawbacks of each and in particular to validate the completeness of sets of partial indicators (42).

This leads to the second consideration for constructing welfare assessments, which is that measures need to be valid. That is, they need to be tracking the actual welfare of the animals, and not something else, such as productivity. If we take the affective state view on welfare, this means that the indicators used within an assessment framework should be linked to the affective states of the animal. While many physical measures may reasonably do so (e.g., skin damage is likely to cause pain), others should be tested to ensure that they do correlate with subjective experiences (e.g., growth rate might reflect a lot of other factors unrelated to welfare experience). What matters is that these links are explicitly tested to ensure the indicators are really valid measures of what the fish is experiencing. Validation can occur in several different ways. Indicators can be validated for correlation with other already established welfare measures. When such measures are not yet available, as may be the case for measures aimed at representing affective states in fish, validation can proceed through using multiple tests under varying conditions that, according to our best available theories of fish evolutionary ecology and behavioral biology, are likely to increase or decrease their welfare [see (33)]. Each of the measures used in a framework should be validated for their link to welfare, as should the overall outputs of the tool itself, to ensure that the final scores do represent the total welfare of the animals assessed. As I have mentioned for many of the indicators discussed, validation of welfare measures for fish is one of the most pressing evidence gaps in fish welfare assessment.

Third, the measures must be feasible for use within an aquaculture assessment or certification scheme. They should be simple enough to use on-farm, without taking too much time or money to implement. Several writers have discussed the requirements for operational welfare indicators (OWIs) for use on fish farms—that they should be valid, reliable, accurate, cost effective, easy to use, and have a low impact on the animals (19, 38–40, 56, 57, 80). This should also include a preference for non-invasive measures, as invasive measures can cause pain and/or stress to the animal and thus themselves compromise welfare [see (18) for discussion of invasive and non-invasive indicators of fish welfare]. It is obviously the case that no set of indicators will perfectly meet all these requirements, as there will be inevitable trade-offs between the different features of any indicator and the “best” outcome will depend on features of the context (42). While some features, such as validity and accuracy will be necessary requirements for indicators to function at all, other features such as cost effectiveness or ease of use will be pursued only so far as is possible within other constraints. There are some particular limitations arising from aquaculture—such as the difficulty in observing individual fish within larger tanks—that will influence the choices made.

One potential development that may help here is the use of precision livestock farming (PLF). PLF involves the use of digital technologies to monitor animals and potentially alert farmers of any issues. Although there is a growing literature on the causes for caution with these methods (81–84), used well they could represent a promising method for monitoring in aquaculture, as “precision fish farming” (85). It is often difficult to monitor groups of fish, as the groups are large and many are underwater where they cannot be directly observed by humans. Use of underwater cameras, infrared, and sonar, alongside computer coding and individual recognition technologies are already commonly used in aquaculture, to monitor feeding for example, and could help identify health and behavioral problems that can't always be seen from the surface (18, 85, 86). In particular, changes in behavior such as aggression or stereotypies could be direct indicators of psychological stress. Video footage could also be used for performing QBA, as described above.

Biosensors or biologgers attached to the fish have been used to directly measure physiological variables such as heart rate or glucose levels (18, 87, 88); that can be used as measures of arousal or physiological stress, potentially useful alongside other more direct measures of the valence of the animals' experiences. These are an invasive method at application, with the corresponding stress of capture for implantation and removal if necessary, but once they are placed the ongoing data collection and monitoring is non-invasive [though effects on growth rate and behavior have been observed; (88)] and may be useful if methods were to be improved to minimize the impact on the fish. The limitations of all these digital methods are the potential costs involved in purchasing and setting up the technology, and training on its use. However, with work already underway testing their use in an aquacultural setting (85), the hope is that this can lead to availability of options that are both affordable and feasible in this context.

Finally, there is the issue of setting the thresholds for acceptable welfare. These thresholds are used to determine whether or not a particular condition, or farming context, sufficiently meets the welfare needs of the animal. These should be reasonable levels set through consultation with multiple stakeholders, including industry professionals, welfare scientists, and fish experts. This first involves agreeing on what level of welfare is considered acceptable for approving or certifying a farm. Ideally, standards for minimum acceptability should be mildly positive, such that the animals are not suffering at all and experience at least some of the positive welfare states that most would consider necessary for a good life. There could also be use of “aspirational” tiers of higher welfare, where animals have a higher positive score. These could potentially be used in consumer labeling schemes to encourage consumers to support high-welfare systems, and to encourage producers to strive for high welfare. The second stage of this process would then be the more research-intensive, involving ongoing testing of each fish species used to find the housing and husbandry conditions that are sufficient for meeting these welfare thresholds.

## 4. Remaining issues

As well as some of the limitations of the current methods of welfare assessment already discussed, there are some more general issues relating to welfare assessment in aquaculture. The first issue is the problem of species diversity. This has been discussed in detail by Franks et al. (89) and Sánchez-Suárez et al. (90). The problem is that the species used in aquaculture are extremely diverse—far from just being “fish”, many of these species are more taxonomically distinct than are terrestrial agricultural animals such as sheep and cows. Just as the same welfare conditions and measurement indicators (inputs and outcomes) are not the same for sheep as they are for cows, neither are they for salmon and tilapia. Instead, sufficient knowledge is required for every species kept, both on their requirements for good housing and husbandry, and the measurement indicators for their welfare. This may prove to be prohibitively difficult and time consuming for all but the most common and well-researched species, such as the salmonids. Certainly, there is currently limited information available on most species.^10^ Filling these information gaps should be a high priority for fish welfare; and approval for aquaculture involving new species should not go ahead where sufficient knowledge is not available, particularly regarding measures for assessing welfare.

There are also issues relating specifically to assessment and certification schemes. It is beyond the scope of this paper to address the design and enforcement of commercial welfare certification, but there are a few considerations I take it are worth keeping in mind for those involved in the design or implementation of these schemes. These are general issues that show up for all current animal welfare certification, but with the relative novelty and expansion of aquaculture, they have particular significance in this context. First, there needs to be clear enforcement, regarding what happens when the conditions are not met—ideally more than just losing certification. Having legislative force behind minimum standards for welfare in aquaculture would help ensure no animals are being kept below an agreed minimum baseline. While enforcement is likely to be easier in countries with existing welfare legislation, we should expect additional challenges for countries still developing in this area. For instance, China produces almost 60% of global aquaculture output (1) but currently has no animal welfare legislation in place. Working with local producers, industry groups, and legislators in these regions could then have a large benefit.

Related to this, there should be transparency behind the requirements, such that they are easily available for consumers and other stakeholders to access. As mentioned in the beginning, many of the current certification schemes are quite opaque, with little detail available on exactly what is required to pass. Available information could take the form of documents listing what is tested and which thresholds need to be met, for those interested in the details. For consumers who are more interested just in ensuring they are buying an ethical product, a simple but clear description of what is achieved relative to the specific needs or wants of the animals could be more helpful. Initiatives such as the British Veterinary Association's “Choose Assured” infographic^11^ represent how this can be successfully achieved. There should also be consistency in the way assessment and certification are applied across different species, or different contexts (such as farms for the same species in different regions). Certification must mean the same thing, or else consumers face difficulty in understanding exactly when they are purchasing high welfare products, diminishing their ability to make this choice. There is a growing concern about “humanewashing”, in which misleading animal welfare certification leads consumers to mistakenly believe they are buying products with higher animal welfare standards than they actually are. Transparency and consistency across labeling schemes would help increase their reliability and trustworthiness (93).

## 5. Conclusion

Aquaculture is a rapidly expanding industry, one that claims to provide many potential global benefits, as a putatively sustainable method of feeding a growing global population. However, it is crucial that animal welfare concerns are central in the development and implementation of aquaculture. If they are not prioritized early on, it becomes much more difficult to adapt in future. To this end, it is important to ensure the availability of high-quality welfare assessment schemes to evaluate the welfare of animals in aquaculture and promote and maintain high welfare standards. In this paper I have discussed some limitations of current methods, and outlined the desirable features for best-practice welfare assessment in aquaculture; particularly relating to the general omission of consideration for subjective welfare experience, and lack of research on the welfare needs and welfare indicators for fish species.

Looking at welfare assessment through this lens identifies some key priorities for research and development of welfare assessment in aquaculture. The first is in altering current welfare assessment and certification frameworks to include some measures that are directly tracking (or attempting to track) how the animals feel. This then brings us to the second priority: increasing research into developing and validating indicators of fish welfare that specifically track affective states—either whole-animal measures such as QBA, or other behavioral or physiological measures that can be demonstrated to indicate how a fish is feeling. Further research is also needed into the best housing and husbandry conditions for different commonly farmed species, with a focus on use of preference and motivation tests to identify what the fishes themselves want, as a guide to what will be best for their welfare. In particular, this should extend to identifying the behavioral needs of the different species, and how to meet them. This will then allow for more empirically-informed decisions about where to set appropriate thresholds for acceptable conditions or welfare levels.

In some cases, such as developing aquaculture in regions without strong animal welfare legislation already in place, these steps may not yet be possible. Instead, we might need to think about the minimum efforts that would be necessary to ensure the worst types of harms are prevented. However, this should not be mistaken for an acceptance that these first steps represent satisfactory fish welfare—they should be seen instead as the “better than nothing” minimal starting points. Primarily, this would be to undertake any type of welfare assessment or certification at all in cases where this might be lacking, even if just using the health-focused frameworks described above. While I have recommended that species not be kept for which specific welfare assessments are lacking, in cases where such species will be used anyway, using some basic or generic health assessment can work as a stop-gap until proper species-specific assessments are developed. Once these are in place, they can work toward adding measures representing how the animals feel, and continuing research into the best housing and husbandry conditions, as described above.

Although this is far from a complete guide to fish welfare assessment, it is my hope that these considerations can be taken on board and used to help improve welfare assessment for aquaculture and, ultimately, to ensure animals used in aquaculture have a higher level of welfare.

## Data availability statement

The original contributions presented in the study are included in the article/supplementary material, further inquiries can be directed to the corresponding author.

## Author contributions

This manuscript was conceived, written, and produced by HB.

## Footnotes

^1^ http://fishcount.org.uk/fish-count-estimates-2/numbers-of-farmed-fish-slaughtered-each-year

^2^ https://bluefood.earth/

^3^Though there are some invertebrate species also used in aquaculture (e.g., octopus, crustaceans), their different behavior, physiology, and welfare needs can raise some distinct considerations. This paper will therefore focus on fish, as these are the only groups for which any commercial welfare assessment schemes currently exist [though see (2) for a detailed assessment tool for zoo-housed cephalopods and decapods], but I take many of the same issues to apply to invertebrates used in aquaculture and development of appropriate welfare assessment is just as important in these cases.

^4^ https://www.bapcertification.org/

^5^ https://www.globalgap.org/uk_en/for-producers/globalg.a.p./integrated-farm-assurance-ifa/aquaculture/

^6^ https://www.asc-aqua.org/

^7^ https://www.rspcaassured.org.uk/farm-animal-welfare/salmon-trout/

^8^Note that the RSPCA previously used animal welfare outcomes for trout, but these were removed from the most recent version of the auditing process; they also state that they are currently developing “Welfare Outcomes Assessments” for salmon, but these are not yet available.

^9^Though it is worth noting that this seems to be entirely possible for fish—for instance, many aquatic species have been shown to respond positively to behavioral and environmental enrichment (49, 50).

^10^If we were to consider other species, such as aquatic invertebrates (e.g., shrimp, octopus, and crayfish), the problem becomes even starker as there are almost no welfare measures currently developed, and development of indicators for these species should be a high priority (91, 92).

^11^ https://www.bva.co.uk/media/3767/bva-uk-farm-assurance-schemes-choose-assured-infographic-october-2020.pdf
